# Lack of Knowledge of HIV Status a Major Barrier to HIV Prevention, Care and Treatment Efforts in Kenya: Results from a Nationally Representative Study

**DOI:** 10.1371/journal.pone.0036797

**Published:** 2012-05-04

**Authors:** Peter Cherutich, Reinhard Kaiser, Jennifer Galbraith, John Williamson, Ray W. Shiraishi, Carol Ngare, Jonathan Mermin, Elizabeth Marum, Rebecca Bunnell, for the KAIS Study Group

**Affiliations:** 1 National AIDS/STI Control Programme (NASCOP), Nairobi, Kenya; 2 Division of Global HIV/AIDS, Center for Global Health, Centers for Disease Control and Prevention, Nairobi, Kenya; 3 Division of Global HIV/AIDS, Center for Global Health, Centers for Disease Control and Prevention, Atlanta, Georgia, United States of America; 4 Division of HIV/AIDS Prevention, National Center for HIV/AIDS, Viral Hepatitis, STD, and TB Prevention, Centers for Disease Control and Prevention, Atlanta, Georgia, United States of America; 5 Division of Global HIV/AIDS, Center for Global Health, Centers for Disease Control and Prevention, Lusaka, Zambia; 6 Division of Adult and Community Health, National Center for Chronic Disease Prevention and Health Promotion, Centers for Disease Control and Prevention, Atlanta, Georgia, United States of America; University of Washington, United States of America

## Abstract

**Background:**

We analyzed HIV testing rates, prevalence of undiagnosed HIV, and predictors of testing in the Kenya AIDS Indicator Survey (KAIS) 2007.

**Methods:**

KAIS was a nationally representative sero-survey that included demographic and behavioral indicators and testing for HIV, HSV-2, syphilis, and CD4 cell counts in the population aged 15–64 years. We used gender-specific multivariable regression models to identify factors independently associated with HIV testing in sexually active persons.

**Results:**

Of 19,840 eligible persons, 80% consented to interviews and blood specimen collection. National HIV prevalence was 7.1% (95% CI 6.5–7.7). Among ever sexually active persons, 27.4% (95% CI 25.6–29.2) of men and 44.2% (95% CI 42.5–46.0) of women reported previous HIV testing. Among HIV-infected persons, 83.6% (95% CI 76.2–91.0) were unaware of their HIV infection. Among sexually active women aged 15–49 years, 48.7% (95% CI 46.8–50.6) had their last HIV test during antenatal care (ANC). In multivariable analyses, the adjusted odds ratio (AOR) for ever HIV testing in women ≥35 versus 15–19 years was 0.2 (95% CI: 0.1–0.3; p<0.0001). Other independent associations with ever HIV testing included urban residence (AOR 1.6, 95% CI: 1.2–2.0; p = 0.0005, women only), highest wealth index versus the four lower quintiles combined (AOR 1.8, 95% CI: 1.3–2.5; p = 0.0006, men only), and an increasing testing trend with higher levels of education. Missed opportunities for testing were identified during general or pregnancy-specific contacts with health facilities; 89% of adults said they would participate in home-based HIV testing.

**Conclusions:**

The vast majority of HIV-infected persons in Kenya are unaware of their HIV status, posing a major barrier to HIV prevention, care and treatment efforts. New approaches to HIV testing provision and education, including home-based testing, may increase coverage. Targeted interventions should involve sexually active men, sexually active women without access to ANC, and rural and disadvantaged populations.

## Introduction

HIV testing and counseling (HTC) is the cornerstone of HIV prevention, care, and treatment. Knowledge of HIV status among HIV-infected persons is associated with a 60% reduction in transmission risk behavior [1], [2]. HTC is an essential component of behavioral interventions [3]–[4] and for targeting specific populations such as HIV discordant couples [5], children [6] or patients with sexually transmitted infections (STI) [7]. HTC is a necessary prerequisite to accessing life-extending care and antiretroviral treatment for persons with HIV infection. Antiretroviral therapy for people with HIV has been associated with a 96% reduction in HIV transmission in discordant couples [8], universal HIV testing and immediate antiretroviral treatment has been suggested as a strategy to control generalized HIV epidemics [9] and would also have a major effect on the HIV-associated tuberculosis epidemic [10].

In spite of HTC's central role in HIV programming, HIV testing coverage remains low in sub-Saharan Africa. Testing coverage documented in population-based surveys in sub-Saharan Africa 2007–2008 ranged from 3.2% in women and 4.9% in men in Liberia to 56.7% in women and 43.0% in men in South Africa [11]. Barriers to HTC vary by setting and stage of the epidemic, but have included low perceived risk [12], stigma and fear of discrimination [13], concerns of confidentiality [14], lack of access to free testing [12], cost of transportation [15], negative perception of testing services [13], shortage of counselors [14] and delays in returning testing results [14].

Kenya has scaled up HTC capacity significantly since 2003, including traditional voluntary counseling and testing sites, mobile, provider-initiated [16] and, more recently, door-to-door HTC [17]. The Kenya AIDS Indicator Survey (KAIS) 2007 was the first nationally representative survey in Kenya that measured laboratory testing results for HIV, herpes simplex virus type 2 (HSV-2), syphilis, and CD4 counts for HIV-infected respondents, and interview data on demographics, sexual behaviors, and service utilization including prior testing history and current HIV status. To improve HTC program planning and delivery, we analyzed KAIS data to compare laboratory testing with self-reported HIV results to determine the prevalence of correct knowledge of HIV status in the country, to identify characteristics of persons aged 15–64 years who had never tested for HIV, and to identify missed opportunities for HIV testing.

## Methods

### Study design

KAIS was a nationally representative, cross-sectional, household sero-survey of persons aged 15 to 64 years conducted from August to December 2007. The survey used a two-stage, stratified sampling design to provide national estimates and separate estimates for urban and rural areas and for each of the eight provinces. The design was comparable with the design of the 2003 Kenya Demographic and Health Survey (DHS), which also included basic questions on HTC. The first stage involved selecting clusters from the same sampling frame that was used for the 2003 DHS, based on the 1999 national census, and the second stage involved the selection of households per cluster with equal probability of selection in the rural-urban strata within each district. Fieldwork was conducted by 29 field teams, each consisting of six data collectors (four interviewers and two laboratory technicians), one supervisor and one driver. Interviewers and laboratory technicians were provided by the Kenya National Bureau of Statistics and the National AIDS/STI Control Programme, respectively. In addition to questionnaires in Kiswahili and English, teams administered questionnaires in local languages where necessary to accommodate respondents that were not conversant in vernacular languages. All questionnaires were back translated into English. Survey personnel participated in intensive two-week training in KAIS procedures, including finger stick, specimen collection for HIV testing and HIV education and counseling. All participants had the option to receive their results individually or as a couple at a nearby referral site [18]. Demographic and HIV/AIDS-specific indicators included use of HIV testing services, HIV status, pregnancy status in women, male circumcision, perception of HIV risk, history of sexually transmitted infections, sexual risk behaviors, and use of in- and outpatient services. Participants were asked ‘Have you ever been tested to see if you have the virus that causes AIDS?’ and if they responded ‘yes’ then the interviewer continued asking ‘When was the last time you were tested?’, ‘Did you get the result of that test?’, and if the participant confirmed that he/she had received the result he/she was asked ‘Would you be willing to share with me the result of your (last) HIV test?’ and ‘Did the test show that you had the HIV virus?’ Further details of KAIS methods are available elsewhere [18].

### Laboratory methods

Blood specimens obtained in households were tested at Kenya's national reference laboratory in Nairobi for HIV, HSV-2, syphilis and CD4 count for HIV-infected persons. HIV testing was performed in a serial testing algorithm by using Vironostika HIV Uni-Form II antigen/antibody (BioMérieux Bv, Boseind, Netherlands) and Murex HIV antigen/antibody (Abbott/Murex-Biotech Ltd, Kent, UK) tests for HIV screening and confirmation, respectively. HIV discrepant specimens were retested with the two assays and polymerase chain reaction (PCR) (Roche HIV DNA v 1.5) tests were conducted on all samples with two sets of discrepant results. For quality control, all positive specimens and a random sample of 5% of negative specimens were retested in a different laboratory using the same testing algorithm. The Kalon HSV Type 2- specific IgG EIA was used for HSV-2 testing. This was a recombinant type-2 antigen (gG2) modified to eliminate reactivity arising from HSV type 1 infection and at the same time retaining the natural antigenic characteristics of HSV-2. For syphilis testing, the *Treponema pallidum* particle agglutination (TPPA) assay was used as a screening test and rapid plasma reagin (RPR) for confirmation.

### Measures

Correct knowledge of HIV status was defined as reported HIV status validated by laboratory testing during the survey. Ever testing was defined as self-report of one or more HIV tests prior to the survey. We developed gender-specific models to assess factors associated with ever having been tested for HIV in ever sexually active persons. Predictor variables included socio-demographic characteristics, pregnancy history and status, HSV-2 infection, syphilis infection, perception of HIV risk, lifetime sexual partners, condom use at last sex, number of outpatient visits in the last 12 months, number of hospitalizations in the last 12 months (excluding outpatient or antenatal care visits), and male circumcision status. We reported predictors for men and women separately because of differences in testing rates. Wealth was defined using a DHS standard composite index of the living standard of a household, calculated using data on a household's ownership of selected assets, materials used for housing construction, water access and sanitation facilities [19]. The wealth index placed households on a continuous scale of relative wealth using principal components analysis. Individuals were categorized according to the score of their household and the sample was divided into quintiles, each with an equal number of individuals, ranging from the poorest to wealthiest.

### Statistical analysis

We performed all analyses in SAS version 9.2 (SAS Institute Inc., Cary, North Carolina, USA) using the sample survey procedures to take into account the sampling structure (stratification, sample weighting, and clustering), and with appropriate domain analysis for each subpopulation of interest. Statistical significance in cross-tabulations was assessed based on Rao-Scott chi-square p-values. The p-value for the difference between CD4 cell counts was calculated using linear regression (PROC SURVEYREG) on log-transformed CD4 counts. Using multivariable logistic regression (PROC SURVEYLOGISTIC), we calculated adjusted odds ratios (AOR) and 95% confidence intervals (CI) to identify variables independently associated with the outcome. Analyses accounted for the stratified cluster design of the survey. Each response was weighted to account for its sampling probability and to adjust for non-response rates. Variables with a p-value <0.1 in bivariate analyses were selected for final multivariable models and backwards elimination was used if they did not remain significant at a 0.05 p-value level. The category with the strongest association was defined as the maximum negative or positive difference from the reference 1. Two-way interactions between variables were considered. Population estimates were calculated based on the 2007 projected Kenyan population [20]. The gap between Kenya's national testing target and testing coverage documented in KAIS was calculated by subtracting 2007 testing rates from Kenya's national target of 80% of the sexually active population to know their HIV status [21] and multiplying with the projected base population. Uncertainty bounds were calculated by multiplying lower and upper confidence limits, respectively, from KAIS estimates for sexual activity and HIV testing.

### Ethics statement

Oral informed consent was obtained from all eligible persons in a three-stage-process: 1) to be interviewed, 2) to have a blood specimen drawn, and 3) to have their blood stored without identifiers for possible future tests. For each of the components, the interviewer signed the consent form to indicate whether or not consent was given. For participants aged 15–17 years, parental or guardian permission was obtained. Eligible persons aged 15–17 years were then asked for their assent. Mature minors, operationalized in KAIS as persons who were married, pregnant or parents, or who were guardians of children aged 0–4 years whose mother died or was HIV infected, did not need parental consent [22]. Investigators obtained a waiver of documentation of informed consent for all participants because the research presented no more than minimal risk of harm to the subjects, the waiver did not adversely affect the rights and welfare of the participants, and the survey involved no procedures for which written consent is normally required outside the research context in Kenya. Survey protocols, including consent procedures, were approved by the Ethics Review Committee of the Kenya Medical Research Institute (KEMRI) and the Institutional Review Board of the U.S. Centers for Disease Control and Prevention (CDC).

## Results

A total of 15,853 of 19,840 eligible persons aged 15–64 years participated in interviews and blood specimen collection, representing a response rate of 80% (women 83%, men 77%). Among those, 1104 or 7.1% (95% CI 6.5–7.7) were HIV infected (men 5.4%, 95% CI 4.7–6.0; women: 8.4%, 95% CI 7.5–9.2). HIV prevalence ranged between 0.8% (95% CI 0–1.6) in North-Eastern to 8.8% (95% CI 6.3–11.4) in Nairobi and 14.9% (95% CI 13.1–16.6) in Nyanza province. Among participants in interviews and blood draws, 87.8% (95% CI 87.1–88.5) reported that they had ever been sexually active (men 85.9%, 95% CI 84.7–87.2; women 89.2%, 95% CI 88.4–90.0). Among those ever sexually active, 27.4% (95% CI 25.6–29.2) of men and 44.2% (95% CI 42.5–46.0) of women reported that they had ever tested for HIV. The gap between Kenya's national testing target and testing coverage documented in KAIS in 2007 was an estimated 4,366,000 (uncertainty bounds 4,280,000–4,452,000) men aged 15–64 years old and 3,295,000 (uncertainty bounds 3,157,000–3,421,000) women.

### Predictors of HIV testing

There were no significant differences between HIV-infected and HIV-uninfected persons regarding HIV testing history. Highest ever HIV testing rates in men were found for age 30–34 years, urban residence, Nairobi province, secondary or higher education, highest wealth index quintile and contact with health facilities. Women who had had an HIV test had similar characteristics as men, but had a highest testing rate at age 20–24 (Table 1). HIV testing rates were significantly higher among women of reproductive age (15–49 years) (49.4%, 95% CI 47.5–51.2) compared to men in the same age group (28.9%, 95% CI 27.1–30.7, p<0.0001). Among women aged 15–49 years who reported ever having sex, 33.5% (95% CI 31.8–35.3) had never been tested for HIV, 48.7% (95% CI 46.8–50.6) had their last HIV test during antenatal care, and an additional 17.7% (95% CI 16.3–19.1) had their last HIV test elsewhere. Among women 15–49 who had ever been tested for HIV, 66.1% (63.6–68.6) had their last HIV test as part of routine antenatal care (ANC). Among older adults (aged 50–64 years), HIV testing was significantly lower overall and higher in men (20.8%, 95% CI 17.7–23.9) compared to women (13.6%, 95% CI 11.0–16.1, p<0.0001).

**Table 1:** Socio-demographic characteristics of persons aged 15–64 years reporting ever HIV testing, by gender, Kenya AIDS Indicator Survey 2007.

	Male				Female			
	N*	n Ever tested	% Ever tested	95% confidence interval	N*	n Evertested	% Ever tested	95% confidence interval
**Characteristics**								
**Total**	5824	1622	27.4	25.6–29.2	7841	3466	44.2	42.5–46.0
**Age**								
15–19	507	84	16.4	12.3–20.4	576	258	45.0	40.5–49.5
20–24	867	290	32.5	29.2–35.9	1385	903	66.8	63.4–70.2
25–29	818	285	32.7	28.4–37.0	1277	768	61.5	57.9–65.1
30–34	753	265	35.6	31.1–40.1	1107	592	52.4	48.2–56.7
35–39	666	207	30.4	25.7–35.1	923	401	41.8	37.9–45.6
40–44	567	148	26.3	22.1–30.5	718	235	30.9	26.2–35.5
45–49	539	127	21.2	17.3–25.0	708	158	20.9	17.3–24.5
50–54	415	98	24.2	19.6–28.9	500	81	16.7	12.9–20.5
55–59	373	70	19.1	13.8–24.4	414	56	15.0	9.8–20.3
60–64	319	48	18.6	11.8–25.3	233	14	5.4	2.3–8.5
**Residence**								
Urban	1535	652	42.1	38.7–45.6	1983	1209	60.5	56.3–64.8
Rural	4289	970	23.0	21.1–25.0	5858	2257	39.0	37.0–41.0
**Region**								
Nairobi	724	362	47.1	42.9–51.3	901	606	68.5	64.1–73.0
Central	860	229	26.7	23.4–30.0	1120	516	46.5	42.9–50.0
Coast	695	198	31.1	25.6–36.7	935	461	50.1	45.1–55.0
Eastern	917	183	19.4	16.6–22.3	1291	456	36.0	33.2–38.7
North Eastern	185	14	6.1	0.4–11.7	235	26	9.3	2.2–16.3
Nyanza	857	255	31.8	27.6–35.9	1231	513	43.6	39.1–47.7
Rift	863	210	24.8	19.2–30.3	1123	453	39.0	34.1–44.0
Western	723	171	23.7	19.8–27.7	1005	435	43.6	39.1–48.0
**Marital Status**								
Currently married/cohabitating	3899	1088	28.1	25.8–30.3	5492	2498	45.6	43.4–47.7
Never married/cohabitating	1557	433	26.0	23.5–28.6	1085	493	44.1	40.7–47.5
Divorced/separated	281	73	25.9	20.2–31.5	594	282	50.4	45.2–55.6
Widowed	87	28	28.3	17.0–39.6	670	193	28.7	24.6–32.9
**Education**								
No education	471	30	7.2	3.7–10.6	1347	262	20.1	16.9–23.3
Incomplete primary	1473	269	18.1	15.3–20.9	2237	904	39.1	36.3–41.9
Complete Primary	1525	391	26.1	23.5–28.8	2033	1019	50.3	47.6–53.0
Secondary or more	2355	932	37.7	35.4–40.0	2224	1281	56.4	53.6–59.1
**Wealth Index**								
Lowest-fourth	4231	914	21.9	20.2–23.5	5869	2269	38.8	36.8–40.8
Highest	1593	708	43.2	39.7–46.7	1972	1197	59.7	57.0–62.4
**Ever pregnant**								
No					832	325	38.9	34.9–42.9
Yes					7009	3141	44.8	42.9–46.7
**Currently pregnant**								
No					6079	2973	48.4	46.5–50.3
Yes					508	298	60.6	55.6–65.6
**Male circumcision**								
No	776	236	30.0	25.7–34.3				
Yes	5033	1384	27.0	25.2–28.9				
**Syphilis infection**								
No	5656	1571	27.4	25.6–29.2	7615	3361	44.3	42.5–46.0
Yes	117	35	28.5	19.2–37.8	137	55	37.9	27.1–48.6
**HSV-2 infection**								
No	4118	1090	26.2	24.3–28.2	4230	1914	46.3	44.3–48.4
Yes	1663	520	30.4	27.7–33.2	3531	1508	41.6	39.3–44.0
**Perceived HIV risk**								
None	1464	393	28.4	25.0–31.7	1457	717	52.0	48.4–55.7
Low	2508	750	28.6	26.0–31.1	2541	1204	46.6	43.5–49.7
Moderate	576	159	28.8	23.7–33.9	1040	486	45.4	41.5–49.3
Great	228	74	31.1	24.2–38.0	573	280	49.1	43.7–54.4
**Lifetime sexual partners**								
1	776	174	23.9	19.8–28.0	3141	1303	42.7	40.2–45.1
2–3	2869	813	28.1	25.9–30.3	4310	2011	46.1	44.1–48.1
4 or more	1806	528	28.0	25.1–31.0	262	104	35.3	28.4–42.2
**Condom use at last sex**								
No	3018	424	14.9	12.9–16.9	4533	1670	37.0	35.0–39.1
Yes	645	161	25.1	21.1–29.2	359	146	45.3	38.7–51.9
**Number of outpatient visits in the last 12 months**								
0	5029	1345	26.4	24.6–28.1	6454	2878	45.0	43.1–46.8
1 or more	775	271	33.7	29.4–37.9	1360	581	41.2	37.5–45.0
**Number of hospitalizations in the last 12 months**								
0	5761	1591	27.2	25.5–29.0	7653	3348	43.7	42.0–45.5
1 or more	63	31	44.6	31.0–58.2	188	118	63.7	55.5–71.9

* Sample size varies slightly across variables due to missing data.

Among ever sexually active adults aged 15–64 years who had never been tested for HIV, 39.6% (95% CI 37.2–41.9) of women and 48.9% (95% CI 46.7–51.1) of men reported they had not been tested because they perceived themselves to be at low risk for HIV infection; 26.7% (95% CI 24.2–29.2) of women and 20.7% (95% CI 18.8–22.5) of men provided no reason for never having been tested. Less than 10% of ever sexually active respondents reported lack of access to testing, fear of others knowing about the test result, not knowing where to go to get tested or lack of access to treatment as reasons for not getting tested.

Table 2 presents results of bivariate and multivariable analyses for variables associated with ever HIV testing in separate models of ever sexually active men and women aged 15–64 years. In final multivariable models, independent factors for testing in men included older age, with a strong association when comparing 25–34 year olds and 15–19 year olds (adjusted odds ratio [AOR] 2.7, 95% CI: 1.6–4.5; p = 0.0002), province with the strongest association North-Eastern versus Nairobi (AOR 0.2, 95% CI: 0.1–0.7; p = 0.0102), education with the strongest association secondary or more versus no primary education (AOR 4.4, 95% CI: 2.2–8.9; p<0.0001), highest wealth index versus the four lower categories combined (AOR 1.8, 95% CI: 1.3–2.5; p = 0.0006), condom use at last sex (AOR 1.6, 95% CI: 1.2–2.2; p = 0.0005) and 1 or more versus 0 outpatient visits in the last 12 months (AOR 1.9, 95% CI: 1.4–2.5; p<0.0001). Independent factors for HIV testing in women included age with the strongest association age 35 and older versus 15–19 years (AOR 0.2, 95% CI: 0.1–0.3; p<0.0001), urban versus rural (AOR 1.6, 95% CI: 1.2–2.0; p = 0.0005), province with the strongest association North-Eastern versus Nairobi (AOR 0.1, 95% CI: 0–0.3; p<0.0001, marital status with the strongest association divorced or separated versus never married or cohabitating (AOR 1.9, 95% CI: 1.2–2.9; p = 0.0035), education with the strongest association secondary or more versus no primary education (AOR 3.9, 95% CI: 2.9–5.3; p<0.0001), ever pregnant (AOR 3.0, 95% CI: 2.2–3.9; p<0.0001), perceived HIV risk with the strongest associations low versus no perceived risk (AOR 0.7, 95% CI: 0.6–0.9; p = 0.0035) and moderate versus no perceived risk (AOR 0.7, 95% CI: 0.6–0.9; p = 0.0007), and 1 or more versus 0 hospitalizations in the last 12 months (AOR 2.4, 95% CI: 1.6–3.7; p<0.0001) (Table 2). While adjusted odds for ever HIV testing in men were 2–3 times as high in all age categories 20 years and older compared to men aged 15–19 years, the adjusted odds in women 35 years or older were only one fifth compared to the reference group. There was a trend of increasing AOR for ever HIV testing with higher levels of education in men and women.

**Table 2:** Unadjusted* and adjusted odds ratios for factors associated with ever testing for HIV from gender-specific models, Kenya AIDS Indicator Survey 2007.

	Male				Female			
Variables	OR (95% CI)**	*p*	AOR*** (95% CI)	*p*	OR (95% CI)	*p*	AOR (95% CI)	*p*
**Age**								
15–19	Reference		Reference		Reference		Reference	
20–24	2.5 (1.8–3.4)	<.0001	2.2 (1.3–3.6)	0.0023	2.5 (1.9–3.1)	<.0001	1.4 (1.0–1.9)	0.0423
25–34	2.6 (1.9–3.6)	<.0001	2.7 (1.6–4.5)	0.0002	1.6 (1.3–2.0)	<.0001	0.8 (0.6–1.0)	0.0728
35 or more	1.6 (1.2–2.2)	<.0001	2.0 (1.2–3.3)	0.0099	0.4 (0.4–0.5)	<.0001	0.2 (0.1–0.3)	<.0001
**Residence**								
Rural	Reference				Reference		Reference	
Urban	2.4 (2.0–2.9)	<.0001			2.4 (2.0–2.9)	<.0001	1.6 (1.2–2.0)	0.0005
**Province**								
Nairobi	Reference		Reference		Reference		Reference	
Central	0.4 (0.3–0.5)	<.0001	0.7 (0.4–1.0)	0.0767	0.4 (0.3–0.5)	<.0001	0.7 (0.5–1.0)	0.0587
Coast	0.5 (0.4–0.7)	<.0001	1.5 (1.0–2.5)	0.0637	0.5 (0.3–0.6)	<.0001	1.0 (0.7–1.6)	0.8447
Eastern	0.3 (0.2–0.3)	<.0001	0.8 (0.5–1.3)	0.3184	0.3 (0.2–0.3)	<.0001	0.5 (0.3–0.8)	0.0012
North Eastern	0.1 (0–0.2)	<.0001	0.2 (0.1–0.7)	0.0102	0 (0–0.1)	<.0001	0.1 (0–0.3)	<.0001
Nyanza	0.5 (0.4–0.7)	<.0001	1.5 (0.9–2.4)	0.1152	0.4 (0.3–0.5)	<.0001	0.6 (0.4–0.8)	0.0048
Rift Valley	0.4 (0.3–0.5)	<.0001	1.1 (0.6–2.0)	0.8310	0.3 (0.2–0.4)	<.0001	0.5 (0.3–0.8)	0.0013
Western	0.4 (0.3–0.5)	<.0001	1.2 (0.7–2.0)	0.5309	0.4 (0.3–0.5)	<.0001	0.6 (0.4–0.9)	0.0268
**Marital Status**								
Never married/cohabitating					Reference		Reference	
Currently married/cohabitating					1.1 (0.9–1.2)	0.4800	1.8 (1.3, 2.3)	<.0001
Divorced/Separated					1.3 (1.0–1.6)	0.0194	1.9 (1.2–2.9)	0.0035
Widowed					0.5 (0.4–0.6)	<.0001	2.6 (1.4–4.9)	0.0028
**Education**								
No primary			Reference		Reference		Reference	
Incomplete Primary	2.9 (1.7–4.7)	<.0001	2.5 (1.2–5.1)	0.0117	2.5 (2.0–3.2)	<.0001	2.1 (1.5–2.8)	<.0001
Complete Primary	4.6 (2.7–7.8)	<.0001	3.5 (1.7–7.1)	0.0007	4.0 (3.3–4.9)	<.0001	2.9 (2.1–3.8)	<.0001
Secondary or more	7.8 (4.6–13.2)	<.0001	4.4 (2.2–8.9)	<.0001	5.1 (4.1–6.4)	<.0001	3.9 (2.9–5.3)	<.0001
**Wealth Index**								
Lowest fourth			Reference		Reference			
Highest	2.7 (2.3–3.2)	<.0001	1.8 (1.3–2.5)	0.0006	2.3 (2.0–2.7)	<.0001		
**Ever pregnant**								
No					Reference		Reference	
Yes					1.3 (1.1–1.5)	0.0101	3.0 (2.2–3.9)	<.0001
**Currently pregnant**								
No					Reference			
Yes					1.6 (1.3–2.0)	<.0001		
**HSV-2 infection**								
No	Reference				Reference			
Yes	1.2 (1.1–1.4)	0.0054			0.8 (0.7–0.9)	0.0005		
**Perceived HIV risk**								
None					Reference		Reference	
Low					0.8 (0.7–1.0)	0.0317	0.7 (0.6–0.9)	0.0035
Moderate					0.8 (0.6–1.0)	0.0151	0.7 (0.6–0.9)	0.0007
Great					0.9 (0.7–1.1)	0.3454	0.9 (0.7–1.1)	0.2802
**Lifetime sexual partners**								
1	Reference				Reference			
2–3	1.2 (1.0–1.6)	0.0683			1.2 (1.0–1.3)	0.0121		
4 or more	1.2 (1.0–1.6)	0.0836			0.7 (0.5–1.0)	0.0498		
**Condom use at last sex**								
No	Reference		Reference		Reference			
Yes	1.9 (1.5–2.4)	<.0001	1.6 (1.2–2.2)	0.0005	1.4 (1.1–1.9)	0.0202		
**Number of outpatient visits in the last 12 months**								
0	Reference		Reference		Reference			
1 or more	1.4 (1.2–1.7)	0.0003	1.9 (1.4–2.5)	<.0001	0.9 (0.7–1.0)	0.0622		
**Number of hospitalizations in the last 12 months**								
0	Reference				Reference		Reference	
1 or more	2.2 (1.2–3.7)	0.0059			2.3 (1.6–3.2)	<.0001	2.4 (1.6–3.7)	<.0001

* Table includes all variables of which at least one category was significantly associated with ever testing for HIV in the bivariate analysis (p<0.01).

** OR = odds ratio, CI = confidence interval.

*** AOR = adjusted odds ratio.

### Awareness of HIV status and testing history among HIV-infected persons

**Figure 1:**
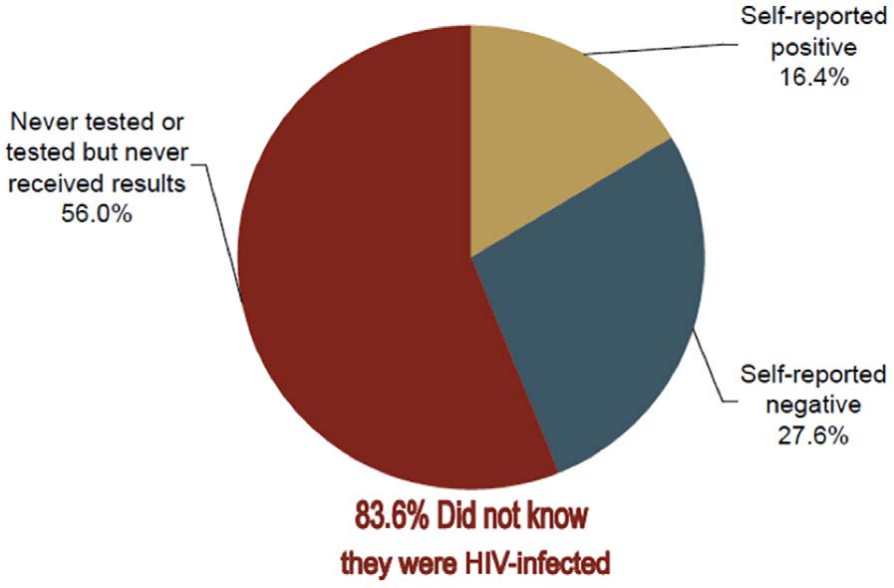
Awareness of HIV status and testing history among HIV-infected persons, Kenya AIDS Indicator Survey 2007.

Among all laboratory-confirmed HIV-infected persons in KAIS 2007, 56.0% (95% CI 51.9–60.0) had never been tested, 27.6% (95% CI 24.3–31.0) of HIV-infected persons reported not to be infected based on their last HIV test, and 16.4% (95% CI 13.2–19.6) reported being infected based on the results of their last HIV test. HIV-infected women (31.4%, 95% CI 27.5–35.4) were significantly more likely than men (19.5%, 95% CI 14.6–24.4, p<0.0001) to self-report negative based on their last test result. In total, 83.6% (95% CI 76.2–91.0) of HIV-infected adults aged 15–64 years were unaware of their HIV infection (Figure 1). Based on these findings, in 2007, an estimated 428,000 (95% CI 371,000–486,000) HIV-infected men and 697,000 (95% CI 626,000–768,000) HIV-infected women nationwide were unaware of their HIV status.

Among laboratory-confirmed HIV-infected persons who reported that they had received a negative HIV test before KAIS 2007, 56.8% (95% CI 50.3–63.3) reported that their last HIV test was performed over 12 months prior to the survey, suggesting that these persons may have been exposed and infected since their last negative test. In addition, the median CD4 cell count was significantly higher in this group (595.0 cells/µL) suggesting possible recent infection, as compared to HIV-infected respondents who also reported that they were positive (412.0 cells/µL, p<0.0001).

### Missed opportunities for HIV testing

**Figure 2:**
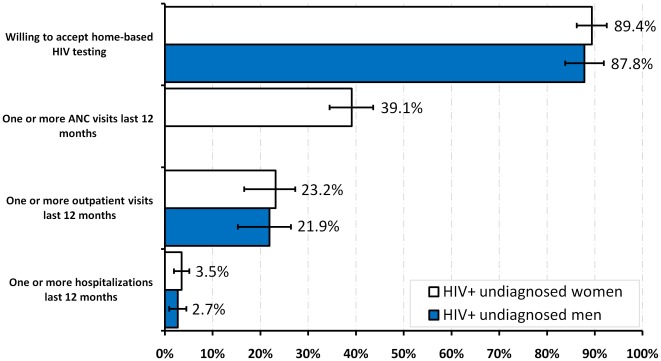
Percentages and 95% confidence intervals of missed opportunities for HIV testing among ever sexually active, HIV-infected undiagnosed persons aged 15–64 years who reported never having been tested for HIV, by gender, Kenya AIDS Indicator Survey 2007.

HIV-infected, undiagnosed women who had never been tested for HIV had the highest proportion of missed testing opportunities during an ANC visit in the 12 months before the survey (39.1%, 95% CI 34.5–43.6) (Figure 2). In 2007, 23.2% (95% CI 17.2–29.2) of men and 45.5% (95% CI 39.7–51.4) of women had at least one missed opportunity for testing during an inpatient, outpatient or ANC visit. Overall, 87.8% (95% CI 83.8–92.0) of men and 89.4% (95% CI 86.2–92.5) of women said that they were willing to be tested at home.

## Discussion

KAIS showed that only one-quarter of ever sexually active men and less than half of ever sexually active women aged 15–64 years in Kenya in 2007 had been tested for HIV. Furthermore, more than 80% of HIV-infected adults aged 15–64 years were unaware of their HIV infection, and among those, one-third reported being uninfected but had a laboratory-confirmed positive HIV test. Time since last HIV test and higher CD4 counts suggests that many of these had sero-converted since their last HIV test.

Considering that in 2003, only 15% of all Kenyans aged 15–49 years had ever been tested (compared to 37% of that age group in KAIS), Kenya made substantial progress in expanding testing [16], [23]. However, overall testing coverage in 2007 remained far below Kenya's national goal of testing 80% of all adolescents and adults [21], and gender difference in testing rates demonstrated a need for special efforts to bring HIV testing to men. Higher testing rates in women of reproductive age and the finding that half of these women reported that they their last HIV test was during antenatal care confirmed the importance of ANC services for HIV testing in women but also raised the question of sufficient access to HIV testing for women who do not get pregnant and older women who are less likely to get HIV tested during ANC. In addition, the large proportion of people with HIV infection reporting a previously negative HIV test indicates ongoing incident infection and the need for more frequent HIV testing than once in a lifetime.

Testing rates for both men and women were higher in urban areas (highest in the capital Nairobi), in better-educated and wealthier persons, and in persons who had contact with health facilities. In multivariable analyses, variables that were independently associated with ever HIV testing for both men and women included age, province, education and contact to health services (outpatient visits in men, hospitalizations in women). In men only, wealth index and condom use at last sex were independently associated with ever HIV testing. In women only, marital status, ever having been pregnant, and perceived HIV risk were independently associated with ever HIV testing. The considerably lower odds for ever testing in women aged 35 years and older compared to men of that age confirmed that women are testing at younger age, most likely driven by ANC services during pregnancy. All provinces with significant AOR were negatively associated with ever HIV testing when compared to Nairobi. This included North-Eastern, the province with limited need for HIV testing, given a HIV prevalence of <1%, and Nyanza with a 40% lower odds for ever HIV testing in women yet an HIV prevalence in women of 17% compared to 10% in Nairobi. Nyanza is a mostly rural province on the shore of Lake Victoria with the highest rates for HIV in the country, at least partially due to a low prevalence of male circumcision, while North Eastern province is less populated and ethnic groups in this area traditionally circumcise. Independent associations with ever HIV testing of both education and wealth (men only) in our study highlight linkages between HIV infection, access to services, and socio-economic status [24]. The main reason for not testing for HIV among those never previously tested was low perception of risk, which has been also reported from Uganda [12].

We found that considerable opportunities for testing were missed during general or pregnancy-specific contacts with health facilities. Our study suggests that more than 90% of all persons in Kenya with undiagnosed HIV infection who had never been tested could potentially be identified through a combination of provider-initiated testing and door-to-door testing in high prevalence provinces. However, coverage of door-to-door testing can decrease when family members cannot be reached at home [25].

The 2008 National Guidelines for HIV Testing and Counselling in Kenya promote a diversified approach to reduce the number of missed opportunities for providing HTC including client-initiated, provider-initiated, self-testing, home-based testing and mass HIV testing campaigns [21]. The guidelines call for integration of HTC into other health services to allow for early detection and better health care for persons living with HIV [21]. Our findings show that targeting sexually active men in general, sexually active non-pregnant and older women (e.g. ≥35 years), and rural and disadvantaged populations should be a priority for prevention efforts, as well as increasing general knowledge about HIV risks in a country with a prevalence of 7%. Standardized quality-control measures for HIV testing, partner testing and mutual disclosure of testing results are additional programmatic implications.

In times of increasing restrictions of funding, national strategies need to consider the most cost-efficient interventions. Menzies and colleagues estimated costs and effectiveness of four HTC strategies in Uganda in 2003–2005 [26]. Door-to-door HCT had the lowest cost per client tested ($8.29) and per client who tested for the first time ($9.21) compared to costs of $11.68 and $14.73, respectively, during hospital-based HCT. However, cost per HIV-positive individual identified was considerably higher for door-to-door HCT ($163.93) than for hospital-based HCT ($43.10). Door-to-door HCT was able to reach more clients as couples (21.6%) than hospital-based HCT (3.2%). Although these results may not be entirely transferable to Kenya, this study confirmed that a mixture of different types of HTC facilities will allow contributing to Kenya's national targets of achieving 80% of the sexually active population to know their HIV status [21], maximize preventive effects that have been shown to be strongest among HIV-positive clients and discordant couples [4], and identify as many persons as possible living with HIV but do not know their status. Further operational research is needed to determine the ideal mixture of services for a country like Kenya and the frequency of repeat testing needed for specific risk populations and in high HIV prevalence areas to identify persons with recent infection early and enroll them into care and treatment programmes. Home-based testing was acceptable to over 80% of persons aged 15–64 years in KAIS 2007 and may help achieve the national testing goal.

Program and survey data in Kenya suggest that testing coverage has continued to increase since KAIS. In 2008–9 [27], 40% of men and 57% of women aged 15–49 years in Kenya had been tested for HIV and had received results at least once in their lifetime (up from 26% and 45% for age 15–49 years, respectively, in KAIS 2007). Nevertheless, given persistent incidence [28], on-going provision of testing will be critical for Kenya's HIV prevention, care and treatment efforts. While testing rates continue to increase in the country and this may place Kenya more towards the higher end of testing rates in sub-Saharan Africa [11], consolidated efforts are needed to reach Kenya's national goal and allow all HIV-infected persons access to life saving treatment. Similar proportions of persons unaware of their HIV status as in our study may be found in other countries in Sub-Saharan Africa with a generalized HIV epidemic similar to Kenya's, indicating the need to include both laboratory testing results for HIV and interview data on prior testing history and current HIV status in population-based HIV surveys.

Our study had several limitations. Cross-sectional surveys do not allow for determination of the sequence of events in time. KAIS was not designed to assess HIV testing among high-risk populations, such as sex workers, men who have sex with men, or intravenous drug users (Kenya has increased surveillance efforts for these populations since 2010). Some data were not available from the study, e.g., whether respondents had not tested for HIV because no transport was available to reach a testing site or they were not able to pay for the transport, or whether they had tested as a couple. There is no generally accepted definition of a high HIV prevalence area; therefore, missed opportunities were reported across Kenya without excluding provinces with relatively low prevalence such as North-Eastern. High rates of undiagnosed infection suggest limited coverage of testing services and relatively high incidence; however, they may also partially reflect reporting bias due to misunderstanding of prior results, denial, misreporting, or false-negative test results. Finally, Kenya's population structure with over 40 ethnic groups of considerable cultural differences may have resulted in some differences in self-reporting. The example of reporting bias of HIV results was discussed above. In general, the direction and magnitude of any potential reporting bias is unknown.

In spite of these limitations, by including for the first time questions on HIV status and CD4 count testing among HIV-infected persons, Kenya's nationally representative HIV survey helped inform HIV program planning with unprecedented detail. Our findings illuminated both the high rates of undiagnosed infection throughout Kenya and the clear opportunities for expanding testing coverage to meet the national and 2008 United Nations goals of universal access to HIV prevention, treatment, care and support [29]. Knowledge of HIV status could help protect millions of people from transmitting HIV unknowingly, from suffering unnecessarily from opportunistic infections, and from dying prematurely with no access to treatment. Three decades into an epidemic, which has already claimed more than an estimated 15 million lives in Africa alone [30], the urgency of universal testing access could not be clearer.
